# Gene discovery in *Triatoma infestans*

**DOI:** 10.1186/1756-3305-4-39

**Published:** 2011-03-18

**Authors:** María L Avila, Valeria Tekiel, Georgina Moretti, Soledad Nicosia, Jacqueline Bua, Estela M Lammel, María M Stroppa, Nelia M Gerez de Burgos, Daniel O Sánchez

**Affiliations:** 1 Instituto de Investigaciones Biotecnológicas, Universidad Nacional de San Martín - CONICET. Av. Gral Paz 5445, Edificio 24, B1650KNA, San Martín, Provincia de Buenos Aires, Argentina; 2 Inst. Nac. de Parasitología, Dr. M. Fatala Chaben, ANLIS C.G. Malbrán, Paseo Colón 568, Buenos Aires, Argentina; 3 Dpto de Microb., Parasitol. e Inmunol., Fac. de Medicina, Universidad de Buenos Aires, Buenos Aires, Argentina; 4 Cat. de Bioq. y Biol. Mol., Fac. de Cs. Médicas, Universidad Nacional de Córdoba, Argentina

## Abstract

**Background:**

*Triatoma infestans *is the most relevant vector of Chagas disease in the southern cone of South America. Since its genome has not yet been studied, sequencing of Expressed Sequence Tags (ESTs) is one of the most powerful tools for efficiently identifying large numbers of expressed genes in this insect vector.

**Results:**

In this work, we generated 826 ESTs, resulting in an increase of 47% in the number of ESTs available for *T. infestans*. These ESTs were assembled in 471 unique sequences, 151 of which represent 136 new genes for the Reduviidae family.

**Conclusions:**

Among the putative new genes for the Reduviidae family, we identified and described an interesting subset of genes involved in development and reproduction, which constitute potential targets for insecticide development.

## Background

Chagas disease affects 8 million people, and ~28 million, in 21 endemic countries of Latin America, are at risk of infection [1]. The etiologic agent of Chagas disease is the parasite *Trypanosoma cruzi*, which is mainly transmitted through blood-sucking insect vectors of the Triatominae subfamily, being *Triatoma infestans*, *Rhodnius prolixus*, and *Triatoma dimidiata *the most epidemiologically important vectors. *T. infestans *is currently the main vector in the southern part of South America including regions of northern Argentina, Bolivia, Paraguay, and southern Peru. *R. prolixus*, on the other hand, is distributed in northern South America (mainly in Colombia and Venezuela) and has few foci in Central America. *T. dimidiata*, is present in Central America and Mexico, and also has limited foci in northern South America [2-4].

Due to population migration from Latin America, Chagas disease is becoming an important health issue in North America, Europe and in the western Pacific region [5]. Currently, there are >390,000 individuals infected with *T. cruzi *in non-endemic regions and, accordingly, serological monitoring of blood banks and organ donors as well as additional controls to detect vertical transmission in newborns must be implemented to prevent the spread of the disease.

As there are currently no vaccines available against *T. cruzi *and effective treatments are limited to chemotherapies that exhibit high toxicity, large efforts are being made to implement prevention strategies, such as domestic vector control and improvement of blood bank surveillance [6]. Currently, the main method of vector control is spraying houses with residual insecticides. However, the occurrence of *T. infestans *populations resistant to pyrethroid compounds in the north of Argentina and Bolivia [7,8] poses the alternative of organophosphate insecticides. Unfortunately, these insecticides, although effective, are very toxic and less accepted by the community due to their unpleasant odor [9]. Novel methods of control will undoubtedly require detailed understanding of the molecular biology of Chagas disease vectors. This will provide unique opportunities to develop new vector control tools such as the use of transgenic vectors refractory to pathogens. The identification of trait genes linked to transposable elements could drive those alleles into wild populations and interrupt disease transmission in the long-term[10,11]. Although in the short run, this strategy is not feasible for Chagas disease control due to the need of an intense laboratory and field research, it is, nevertheless, an approach that deserves to be explored.

The availability of complete genome sequences of medically important vectors will accelerate the identification of new target genes to advance in novel vector control strategies, such as the use of transgenic vectors [12], and the control of pathogen transmission by targeting genes responsible for host-seeking behaviors. In line with this, several initiatives to sequence vectors of infectious diseases have been launched and some have been finished [13,14]. In 2005, the NIH-National Human Genome Research Institute (NHGRI) and the Washington University Medical School Genome Sequencing Center decided to sequence the genome of *R. prolixus*, as a model for the Reduviidae family [15]. However, advancing in *T. infestans *genomics is also of major importance, as this is the main vector in many South American areas, particularly in Argentina.

As an alternative or complement of genome projects, transcriptome studies performed by sequencing cDNA libraries of Expressed Sequence Tags (ESTs) or of open reading frame ESTs (ORESTES), constitute a rapid, low-cost and effective way to obtain information of the transcriptionally active regions of any organism and to discover novel genes [16-18].

Currently, transcriptome information available for the *Triatoma *genus is very limited. In fact, only salivary gland ESTs projects have been carried out for *T. brasiliensis*, *T. infestans *and *T. dimidiata *[19-21]. The study of *T. infestans *sialome has provided a set of 1534 salivary gland cDNA sequences, 42% of which encode proteins of a putative secretory nature - most of which (55%) have been described as lipocalins [20].

With the aim to gain further insights into *T. infestans *transcriptome and to discover novel genes in this insect, we started a small project to generate ESTs from this vector. In the present work, we report the analysis of 826 ESTs and provide information of interest for the development of new drugs to target the biological cycle of the insect.

## Results and discussion

### ESTs overview

To generate *T. infestans *ESTs, a total of 1881 clones were sequenced from different cDNA libraries obtained from different tissues and developmental stages (for details see Additional file 1: Table S1). The complete dataset was analyzed as a unit, independently of the library to which the sequences belonged.

After the cleaning steps, 826 sequences (i.e., 44% of all the clones sequenced) were classified as high quality ESTs (Table 1) and were uploaded into the GenBank ESTs database with accession numbers from [GenBank:HO762759 to GenBank:HO763584]. This dataset increases the number of available *T. infestans *ESTs by 47%, from 1738 to 2564.

**Table 1:** Summary of *T. infestans *ESTs.

Description	Number	%
Total number of clones sequenced	1881	100

High quality ESTs	826^a^	44
Contigs	119	
Singlets	352	
Assembled Unique sequences (AUS)	471	25

AUS with positive hits	185^b^	39
BLASTX NR protein database	176	
InterProScan	20	
AUS with no hits	286	61

AUS with EC numbers	44	26
AUS GO annotated	123	26

^a^ESTs deposited in GenBank ESTs database with accession numbers [GenBank:HO762759 to GenBank:HO763584]. ^b^See Additional file 2: Table S2 for hit details.

To unify overlapping high quality ESTs, eliminate redundancies and facilitate further studies, a *de novo *assembly was performed. This step grouped the high quality ESTs in 119 contigs and 352 singlets, summing up to 471 assembled unique sequences (AUS), with an average size of 358 bp, representing different putative transcripts or different parts of the same transcript of *T. infestans *(Table 1).

To know the percentage of new *T. infestans *ESTs provided by the AUS generated, BLASTN searches were performed against the EST database at NCBI. Only 10% (47) of the AUS gave positive hits against *T. infestans*, indicating that the remaining 90% (424) AUS are novel ESTs sequences for *T. infestans *(Additional file 2: Table S2).

BLASTX searches yielded 37% (176) of AUS with significant similarity to protein sequences (E-value < 1e-10) (Table 1). InterProScan searches yielded 4% (20) of AUS with similarity to a protein domain, 45% of which also had a hit in the NR protein database. As a whole, homologies against protein databases summarized 39% (185) of AUS with hits in at least one database (Table 1). Additionally, Enzyme Commission (EC) codes were assigned to 44 AUS, and Gene Ontology (GO) terms to 123 AUS (Table 1).

### Comparative analysis to other taxa

**Figure 1:**
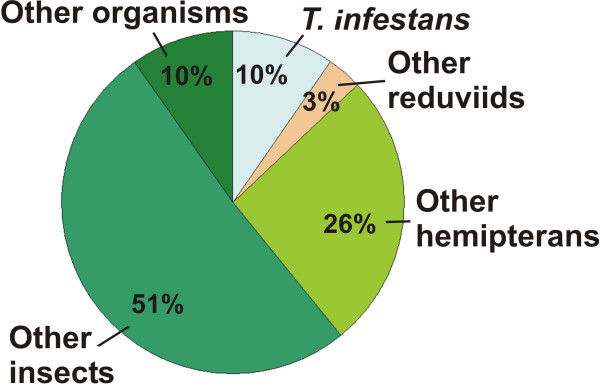
**Number of AUS with top-hits in the different categories**. Results from BLASTX searches in the NR protein database using a cutoff E-value < 1e-10. Percentage values were calculated considering the 176 AUS with BLASTX significant matches as 100%.

The AUS with positive BLASTX results (n = 176, see Table 1) were taxonomically classified according to the top-hit (Figure 1). Only 10% (17) of these AUS were most similar to predicted proteins from *T. infestans *and 3% (6) to predicted proteins from four members of the family Reduviidae (*R. prolixus, T. dimidiata, T. vitticeps and T. brasiliensis*; see Additional file 2: Table S2 for details). These results can be partially explained by the fact that at the time that this paper was written there were no complete genomes available for any triatomine. Actually, the phylogenetically closest sequenced genome available belongs to the hemipteran *Acyrthosiphum pisum*, which has been recently released in a draft assembly status [22]. Not surprisingly, *A. pisum *is the most represented organism in the "other hemiptera" category with 20 top-hits. The most represented organism in the "other insects" category, and in the entire dataset, is the coleopteran *Tribolium castaneum*, with 29 top-hits. This last result is in agreement with the findings for the *A. pisum *genome, for which the organisms with the highest shared gene content were *T. castaneum *and *Nassonia vitripennis*, both with 53% [22].

However, it is expected that more closely related orthologs will begin to appear as genome projects of phylogenetically closer organisms are finished and annotation information becomes available, in particular from the in-progress genome project of *R. prolixus *[23]. Today, this is reflected when using our AUS as queries in BLASTN searches against the WGS database at NCBI, where 56% (265) of the top-hits are from *R. prolixus *whole-shotgun genome sequences (Additional file 2: Table S2). Consequently, our study will contribute with gene prediction and annotation in the aforementioned genome project.

### Gene Ontology annotation

**Figure 2:**
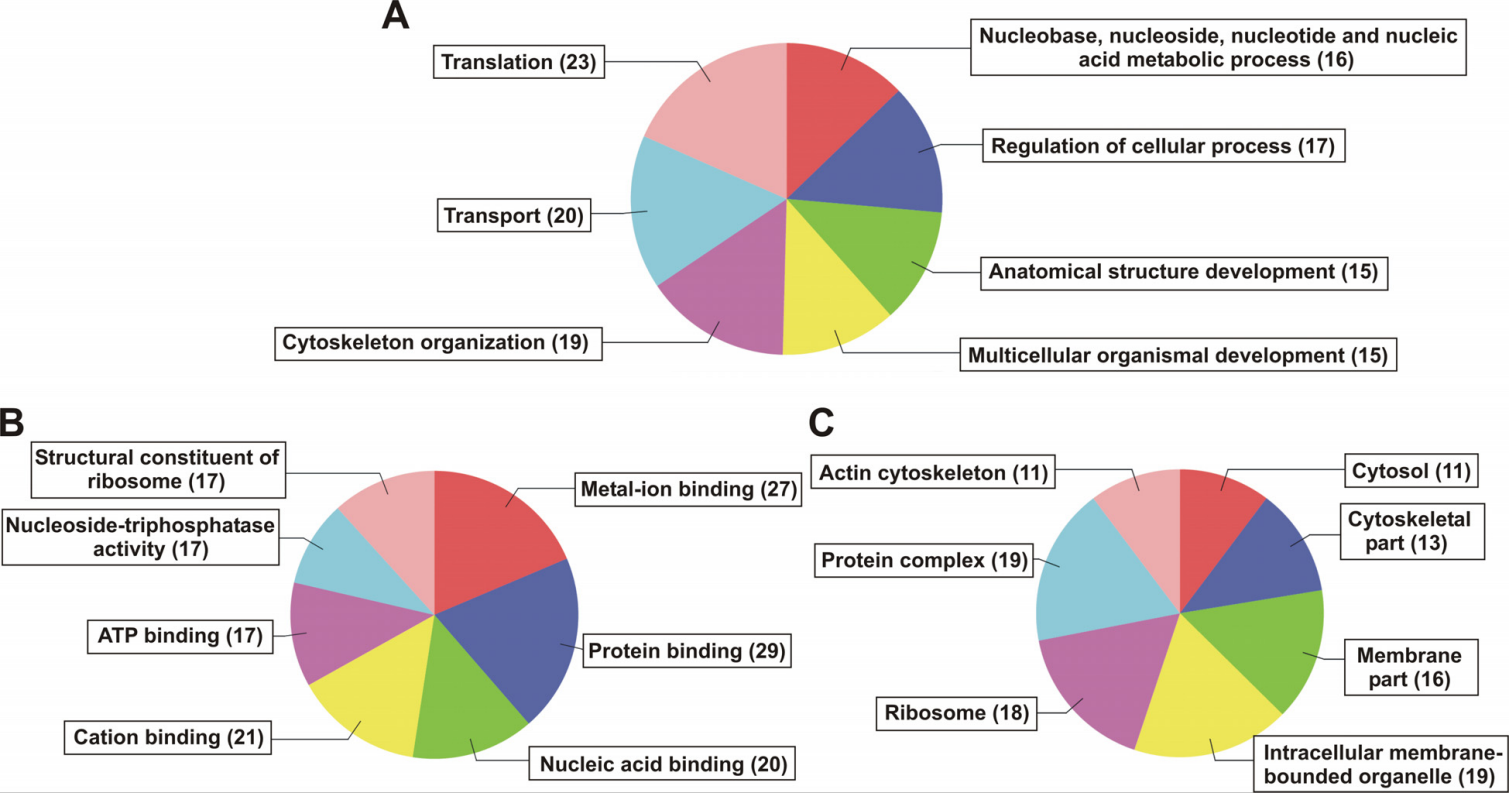
**Most frequently assigned terms of each of the three GO sub-ontologies**. Pie charts only show terms with more than 15, 13 and 10 AUS associated with Biological Process (A), Molecular Function (B) and Cellular Component (C) vocabularies, respectively.

The GO project is a bioinformatics initiative aiming to standardize the representation of gene and gene product attributes across species [24]. GO provides three controlled vocabularies to describe a gene product in terms of: the biological process (BP) in which it is involved, its molecular function (MF), and the cellular component (CC) where it acts. The assignation of GO terms to our dataset with the default parameters resulted in the annotation of 123 AUS. Among them, 95 were classified according to BP, 110 to MF and 78 to CC, and 60 had at least one GO term of each vocabulary. The most frequently assigned terms in each vocabulary are presented in Figure 2. For the BP category, the three most common terms were "translation" (23), "transport" (20) and "cytoskeleton organization" (19) (Figure 2A). For the MF category, the four most common terms were "protein binding" (29), "metal ion binding" (27), "cation binding" (21) and "nucleic acid binding" (20) (Figure 2B); and for the CC category, the most common terms were "protein complex" (19), "intracellular membrane-bounded organelle" (19) and "ribosome" (18) (Figure 2C). Interestingly, when we performed a similar analysis upon the *T. infestans *ESTs already in the EST database, in the BP vocabulary, the term "evasion or tolerance of host defense response" was by far the most represented (data not shown). This result is directly associated with the fact that the *T. infestans *ESTs available up to now are significantly enriched in sequences coding for salivary lipocalins. Thus, we think that the heterogeneity of our ESTs will enlarge and complement the data already available, particularly with regard to genes involved in diverse biological processes.

### Description of genes of interest

In this work we identified 151 AUS that did not match with protein sequences of *T. infestans *or other reduviids and which represent 136 novel genes for the Reduviidae family (Table 2 and Additional file 3: Table S3). A detailed analysis of the putative genes identified is not within the scope of this work and will certainly be carried out by researchers interested in the field. However, a number of interesting matches with sequences from non-reduviid organisms were observed in the category "development and reproduction" (Additional file 3: Table S3). Among them, we found several transcripts encoding proteins related to the juvenile hormone (JH) metabolism such as vitellogenin, hexamerins and JH esterase. The JH is a sesquiterpene essential for development and reproduction in insects [25]. Interestingly, it has been shown that a compound that interferes with the metabolism of the JH induce biomorphological alterations in three species of the Triatominae subfamily, including *T. infestans *[26], suggesting that the JH could be a new target to control the vector population.

**Table 2:** Putative new genes for the Reduviidae family in each category.

New genes	Category
16	Membrane traffic regulation and signal transduction (16 AUS)

22	Nucleic Acid metabolism (25 AUS)

10	Development and reproduction (16 AUS)

14	Protein metabolism (14 AUS)

14	Ribosomal proteins (14 AUS)

6	Cytoeskeleton (9 AUS)

8	Muscle (9 AUS)

7	Cuticle/exoeskeleton (9 AUS)

4	Mitochondrial proteins (6 AUS)

20	Others (20 AUS)

13	Hypothetical, conserved, unknown (13 AUS)

Classification was made taking into account the sequence description of the 151 AUS with BLASTX hits from non-reduviid organisms. For further category details see Additional file 3: Table S3.

ESTs belonging to Contig114 and Contig118, and the singlets Tinf_aii_1_i4 and Tinf_am_4_a4, have significant matches to different parts of the vitellogenin protein (Additional file 3: Table S3). This hemolymph lipoprotein, a predominant yolk protein precursor, is synthesized mainly in adult female fat body, secreted into the hemolymph and taken up by the growing oocytes. This biological process called vitellogenesis allows embryonic development outside the maternal body and is regulated by the JH in most insects [27]. Although vitellogenin has been purified and described biochemically for *T. infestans *[28,29], its gene sequence has not been determined yet, therefore our data will enable and facilitate obtaining the entire gene sequence.

Contig15, Contig51, Tinf_4itla_3_a16 and Tinf_4its_12_A11 putatively code for different hexamerin proteins or different parts of the same hexamerin protein (Additional file 3: Table S3). These proteins are synthesized by the fat body and secreted into the hemolymph where they reach extraordinary concentrations prior to metamorphosis, a process in which they serve as a source of amino acids. They also may function as JH-binding proteins [30,31]. The biosynthetic processes related to the production of the very high density lipoprotein (VHDL), a *T. infestans *hexamerin, have been described by Rimoldi et al. (1997) [32]. In addition, hexamerins have also been described in *R. prolixus *[33] and in *Riptortus clavatus *[34], both members of the panheteroptera hemipteran clade. However, the hemipteran *A. pisum *apparently lacks these proteins [22].

Tinf_4its_8_B10 has significant homology to the JH esterase (JHE) (Additional file 3: Table S3). This carboxylase enzyme, along with the JH epoxide hydrolase, is responsible for JH degradation. It is synthesized mainly by the fat body and released into the hemolymph where its high levels are associated with low levels of circulating JH [35]. Interestingly, recent studies have shown that transgenic silkworms overexpressing JHE from embryonic stages undergo precocious larval-pupal metamorphosis [36]. Due to its key role in the regulation of JH titers during insect development and reproduction, JHE has been the target for the investigation of selective drugs [37,38]. Therefore, this protein seems to be also a promising drug target to control Chagas disease vectors by targeting the biological cycle of the insect.

We also found interesting transcripts representative of proteins involved in development and reproduction but without a direct relationship with JH. For example, the encoded protein in Contig91 is similar to the lingerer protein (Additional file 3: Table S3); the lingerer gene (*lig*) from *Drosophila *has been shown to be involved in the initiation and completion of copulation [39]. Males carrying a hypomorphic mutation in *lig *cannot withdraw their genitalia at the end of copulation. In addition, a severe reduction of the *lig *dosage causes repeated mating attempts without success, and complete loss of *lig *function causes lethality during early pupal stage [39].

Other interesting matched proteins in other insects are the protein Dumpy (Contig72), which is essential for proper wing formation in *Drosophila *[40], and a growth factor (Contig50), which belongs to the family of imaginal disc growth factors (IDGFs). In *Drosophila*, these polypeptide growth factors are expressed most strongly in the embryonic yolk cells and in the fat body of the embryo and larva, and cooperate with insulin to stimulate the proliferation, polarization and motility of imaginal disc cells [41].

## Conclusions

In this work, we generated 826 ESTs from *T. infestans*, which were assembled in 471 unique sequences, 151 of which represent 136 new genes for the Reduviidae family. These data, already available to researchers interested in the field, might provide new insights into the biology of *T. infestans*, and potential targets for future rational drug design to control *T. infestans*, an important vector of Chagas disease in South America.

## Methods

### T. infestans specimens, tissue samples and mRNA isolation

Fourth instar specimens were provided by the Center of Vector Reference, Córdoba, Argentina, and by the National Institute of Parasitology "Dr. Mario Fatala Chaben", Buenos Aires, Argentina. Muscle tissue samples from adult specimens were obtained from the Department of Biochemistry and Molecular Biology, Faculty of Medicine, National University of Córdoba, Argentina. Gut tissue samples from adult specimens were provided by the Department of Microbiology, Parasitology and Immunology, Faculty of Medicine, National University of Buenos Aires, Argentina.

In all cases, total RNA was isolated using TRIzol reagent (Invitrogen) and residual genomic DNA was removed by DNase treatment using RNase-free DNase I (Ambion). Both procedures were performed according to the manufacturer's instructions.

The absence of residual DNA was tested by PCR, using the following primers: CytB_Fwd 5'- GGACAAATATCATGAGGAGCAACAG and CytB_Rev 5'- ATTACTCCTCCTAGCTTATTAGGAATTG, targeted to the mitochondrial cytochrome B gene of *T. infestans *[42], with a final concentration of 3 mM MgCl_2_. PCR conditions were: 94°C 2 min - (94°C 30 sec - 47°C 40 sec - 72°C 50 sec) 30 cycles - 72°C 10 min. DNase untreated samples were included as PCR positive controls. The integrity of the total RNA was assessed by agarose electrophoresis together with the A260/A280 ratio.

Poly(A)+ RNA was purified using the MicroPoly(A)Purist™ Kit (Ambion) or the PolyATtract^® ^mRNA Isolation System IV (Promega), both according to the manufacturer's instructions.

### cDNA synthesis and library construction

Three different strategies were applied for cDNA library construction: i) a conventional library was constructed by using the SMART cDNA Synthesis Kit (Clontech), following the manufacturer's instructions; ii) a second set of conventional libraries was constructed using the PCR-Select™ cDNA Subtraction Kit (Clontech) as unsubtracted libraries, which implies ligating the sample with both adaptors at the same time, and not performing the hybridization steps; and iii) ORESTES libraries were constructed as described by Dias-Neto et al. (2000), with minor modifications [43]. Libraries ii) and iii) were ligated into pGEM-T Easy vector (Promega). All libraries were transformed into *E. coli *DH5-alpha cells.

*T. infestans *specimens, tissue samples and cDNA library construction strategies are summarized in Additional file 1.

### Nucleotide Sequencing

Independent colonies were picked and transferred into 384- or 96-well culture plates containing: 80 μl of 2xTY medium supplemented with 10% of HMFM anti-freezing medium [44] and 0.1% of ampicillin (10 mg/ml) in each well. Colonies were grown at 37°C overnight and stored at -70°C until use. Each colony was assigned with a clone number consisting of the name of the library, followed by the culture plate number and well position.

An aliquot of 15 μl of each culture was transferred into a 96-well PCR microplate, and incubated for 5 minutes at 100°C. A colony-PCR reaction was performed in a new 96-well PCR microplate, using universal M13 forward and reverse primers, and 1 μl of the boiled culture. PCR conditions were: 94°C 2 min - (94°C 30 sec - 55°C 30 sec - 72°C 40 sec) 30 cycles - 72°C 5 min. All PCR reactions were carried out in a T-Professional thermocycler (Biometra).

The amplification products were visualized in a 1.5% agarose gel with ethidium bromide. The reactions that had only one band greater than 300 or 400 bp, depending on the library, were selected for clean-up and sequencing. For clean-up, 3 - 5 μl of the PCR product was incubated with 5 U of Exonuclease I (Fermentas) and 0.5 U of Shrimp Alkaline Phosphatase (Fermentas) (30 min at 37°C and then 15 min at 85°C). Single-pass sequencing was performed on each cleaned template in an ABI 3130 sequencer (Applied Biosystems) using T7 or Sp6 primers, or the primer pDNR-LIB_Forward-53: 5' - TATCAGTCGACGGTACC in the case of the vector pDNR-LIB.

### Data cleaning and assembly

The raw traces obtained were base-called using the Phred program [45] with quality cut-off set at 20. Lucy2 [46] was used to remove the vector and adaptor sequences, as well as to trim low quality regions and poly-A/T tails (>14 contiguous bases). Sequences of less than 100 bp were excluded.

The BLASTN program was used to search for similar sequences through the non-redundant (NR) nucleotide database at National Centre for Biotechnology Information (NCBI) with an E-value < 1e-3. Sequences with significant similarity to Triatoma virus sequences, rRNA or mtDNA genes of any organism were removed. The remaining high quality sequences were assembled with the Contig Assembly Program 3 (CAP3) [47] using the default parameters of 40 bp of overlap length and 80% of overlap identity. Phred, Lucy2 and CAP3 steps were performed with the web-based pipeline for ESTs, EST-piper [48].

### Analysis and annotation of ESTs

The assembled unique sequences (AUS) were used as queries in BLASTN searches against the NCBI Whole-genome shotgun reads (WGS) database with a E-value < 1e-3, and against the EST database with an E-value < 1e-3. Positive *T. infestans *matches in the EST database were considered to be the same sequence when the E-value was lower than 10e-13. All BLASTN searches were carried out with the blastcl3 program, which allows blasting in batch.

To annotate each AUS, homology searches in protein sequence and protein domain databases, EC numbers assignation and GO term mapping were carried out using the Blast2GO software [49] (Additional file 2: Table S2). The BLAST step was carried out using BLASTX with an E-value cutoff of < 1e-10, and a minimal high-scoring segment pair (HSP) alignment length of 20 amino acids. The BLAST description annotation function was based on the first 20 hits that fulfill the E-value cutoff. InterProScan function (from the European Bioinformatics Institute [50]) was used to search in the following protein domain databases: HMM-Pfam, HMM-Smart, HMM-Tigr, ProfileScan, PatternScan, Superfamily and HMM-Panther. AUS were mapped to Enzyme Commission (EC) numbers [51] and to the three Gene Ontology (GO) vocabularies [52] (Biological Process (BP), Molecular Function (MF) and Cellular Component (CC)) and were annotated using default settings, including default Evidence Codes values. For a better interpretation of data, multi-level pie charts were generated for the BP, MF and CC vocabularies, filtering by 15, 13 and 10 sequences respectively.

## Competing interests

The authors declare that they have no competing interests.

## Authors' contributions

DOS design the experiments. MLA, GM, SN, VT and DOS performed the experiments. JB, EML, MMS, NGB contributed with *T. infestans *specimens. MLA did the bioinformatic analysis and wrote the first version of the manuscript. MLA, VT and DOS revised and finalized the manuscript. All authors revised and approved the final version of the manuscript.

## Supplementary Material

Additional file 1 **Table S1**. Summary of *T. infestans *libraries. Click here for file

Additional file 2 **Table S2**. *T. infestans *AUS analyses. Click here for file

Additional file 3 **Table S3**. Putative novel genes for the Reduviidae family. Click here for file

## References

[B1] Rassi A Jr Rassi A Marin-Neto JA Chagas disease Lancet 2010 375 1388 1402 10.1016/S0140-6736(10)60061-X 20399979

[B2] Schofield CJ Jannin J Salvatella R The future of Chagas disease control Trends Parasitol 2006 22 583 588 10.1016/j.pt.2006.09.011 17049308

[B3] Who, how, what and where? Nature 2010 465 n7301_supp S8 S9 10.1038/nature09222 20571555

[B4] Petherick A Country by country Nature 2010 465 n7301_supp S10 S11 10.1038/nature09223 20571547

[B5] Coura JR Vinas PA Chagas disease: a new worldwide challenge Nature 2010 465 n7301_supp S6 S7 10.1038/nature09221 20571554

[B6] Moncayo A Silveira AC Current epidemiological trends for Chagas disease in Latin America and future challenges in epidemiology, surveillance and health policy Mem Inst Oswaldo Cruz 2009 104 Suppl 1 17 30 19753454 10.1590/s0074-02762009000900005

[B7] Lardeux F Depickere S Duchon S Chavez T Insecticide resistance of Triatoma infestans (Hemiptera, Reduviidae) vector of Chagas disease in Bolivia Trop Med Int Health 2010 15 1037 1048 10.1111/j.1365-3156.2010.02573.x 20545921

[B8] Picollo MI Vassena C Santo Orihuela P Barrios S Zaidemberg M Zerba E High resistance to pyrethroid insecticides associated with ineffective field treatments in Triatoma infestans (Hemiptera: Reduviidae) from Northern Argentina J Med Entomol 2005 42 637 642 10.1603/0022-2585(2005)042[0637:HRTPIA]2.0.CO;2 16119553

[B9] Reporte sobre la enfermedad de Chagas http://apps.who.int/tdr/svc/publications/tdr-research-publications/reporte-enfermedad-chagas

[B10] Aksoy S Hao Z Strickler PM What can we hope to gain for trypanosomiasis control from molecular studies on tsetse biology? Kinetoplastid Biol Dis 2002 1 4 10.1186/1475-9292-1-4 12234385 PMC119325

[B11] Toure YT Oduola AM Morel CM The Anopheles gambiae genome: next steps for malaria vector control Trends Parasitol 2004 20 142 149 10.1016/j.pt.2004.01.008 15036036

[B12] Crampton J Morris A Lycett G Warren A Eggleston P Transgenic mosquitoes: a future vector control strategy? Parasitol Today 1990 6 31 36 10.1016/0169-4758(90)90057-B 15463283

[B13] Holt RA Subramanian GM Halpern A Sutton GG Charlab R Nusskern DR Wincker P Clark AG Ribeiro JM Wides R The genome sequence of the malaria mosquito Anopheles gambiae Science 2002 298 129 149 10.1126/science.1076181 12364791

[B14] Nene V Wortman JR Lawson D Haas B Kodira C Tu ZJ Loftus B Xi Z Megy K Grabherr M Genome sequence of Aedes aegypti, a major arbovirus vector Science 2007 316 1718 1723 10.1126/science.1138878 17510324 PMC2868357

[B15] Huebner E The Rhodnius Genome Project: The promises and challenges it affords in our understanding of reduviid biology and their role in Chagas' transmission [abstract] Comp Biochem and Physiol 2007 148 S130 S130 10.1016/j.cbpa.2007.06.325

[B16] Boguski MS Tolstoshev CM Bassett DE Jr Gene discovery in dbEST Science 1994 265 1993 1994 10.1126/science.8091218 8091218

[B17] Nagaraj SH Gasser RB Ranganathan S A hitchhiker's guide to expressed sequence tag (EST) analysis Brief Bioinform 2007 8 6 21 10.1093/bib/bbl015 16772268

[B18] Verdun RE Di Paolo N Urmenyi TP Rondinelli E Frasch AC Sanchez DO Gene discovery through expressed sequence Tag sequencing in Trypanosoma cruzi Infect Immun 1998 66 5393 5398 9784549 10.1128/iai.66.11.5393-5398.1998 PMC108675

[B19] Santos A Ribeiro JM Lehane MJ Gontijo NF Veloso AB Sant'Anna MR Nascimento Araujo R Grisard EC Pereira MH The sialotranscriptome of the blood-sucking bug Triatoma brasiliensis (Hemiptera, Triatominae) Insect Biochem Mol Biol 2007 37 702 712 10.1016/j.ibmb.2007.04.004 17550826 PMC1896098

[B20] Assumpcao TC Francischetti IM Andersen JF Schwarz A Santana JM Ribeiro JM An insight into the sialome of the blood-sucking bug Triatoma infestans, a vector of Chagas' disease Insect Biochem Mol Biol 2008 38 213 232 10.1016/j.ibmb.2007.11.001 18207082 PMC2262853

[B21] Kato H Jochim RC Gomez EA Sakoda R Iwata H Valenzuela JG Hashiguchi Y A repertoire of the dominant transcripts from the salivary glands of the blood-sucking bug, Triatoma dimidiata, a vector of Chagas disease Infect Genet Evol 2010 10 184 191 10.1016/j.meegid.2009.10.012 19900580 PMC2941348

[B22] International_Aphid_Genome_Consortium Genome sequence of the pea aphid Acyrthosiphon pisum PLoS Biol 2010 8 e1000313 10.1371/journal.pbio.1000313 20186266 PMC2826372

[B23] Huebner E The Rhodnius Genome Project: The promises and challenges it affords in our understanding of reduviid biology and their role in Chagas' transmission [abstract] Comp Biochem Physiol 2007 148 Supplement 1 s130

[B24] Gene_Ontology_Consortium The Gene Ontology in 2010: extensions and refinements Nucleic Acids Res 2010 38 D331 335 10.1093/nar/gkp1018 19920128 PMC2808930

[B25] Kort CAD Granger NA Regulation of the Juvenile Hormone Titer Annu Rev Entomol 1981 26 1 28 10.1146/annurev.en.26.010181.000245

[B26] Jurberg J Galvao C Bowers WS Garcia ES Azambuja P Biomorphological alterations induced by an anti-juvenile hormonal compound, 2-(2-ethoxyethoxy)ethyl furfuryl ether, on three species of triatominae larvae (Hemiptera, Reduviidae) Mem Inst Oswaldo Cruz 1997 92 263 268 9332589 10.1590/s0074-02761997000200022

[B27] Atella GC Gondim KC Machado EA Medeiros MN Silva-Neto MA Masuda H Oogenesis and egg development in triatomines: a biochemical approach An Acad Bras Cienc 2005 77 405 430 16127549 10.1590/s0001-37652005000300005

[B28] Rimoldi OJ Soulages JL Gonzalez SM Peluffo RO Brenner RR Purification and properties of the very high density lipoprotein from the hemolymph of adult Triatoma infestans J Lipid Res 1989 30 857 864 2677201

[B29] Salomon OD Stoka A Vitellin and vitellogenin characterization of Triatoma infestans and related species Acta Physiol Pharmacol Latinoam 1986 36 419 429 3111174

[B30] Braun RP Wyatt GR Sequence of the hexameric juvenile hormone-binding protein from the hemolymph of Locusta migratoria J Biol Chem 1996 271 31756 31762 10.1074/jbc.271.49.31756 8940201

[B31] Telfer WH Kunkel JG The function and evolution of insect storage hexamers Annu Rev Entomol 1991 36 205 228 10.1146/annurev.en.36.010191.001225 2006868

[B32] Rimoldi OJ Gonzalez MS Brenner RR Biochemistry of the evolution of Triatoma infestans. XII. Biosynthesis and secretion of a very high density lipoprotein Acta Physiol Pharmacol Ther Latinoam 1997 47 77 86 9339237

[B33] Faria FS Garcia ES Goldenberg S Synthesis of a haemolymph hexamerin by the fat body and testis of Rhodnius prolixus Insect Biochem Mol Biol 1994 24 59 67 10.1016/0965-1748(94)90123-6

[B34] Miura K Shinoda T Yura M Nomura S Kamiya K Yuda M Chinzei Y Two hexameric cyanoprotein subunits from an insect, Riptortus clavatus. Sequence, phylogeny and developmental and juvenile hormone regulation Eur J Biochem 1998 258 929 940 10.1046/j.1432-1327.1998.2580929.x 9990310

[B35] Mackert A do Nascimento AM Bitondi MM Hartfelder K Simoes ZL Identification of a juvenile hormone esterase-like gene in the honey bee, Apis mellifera L.--expression analysis and functional assays Comp Biochem Physiol B Biochem Mol Biol 2008 150 33 44 10.1016/j.cbpb.2008.01.004 18308604

[B36] Tan A Tanaka H Tamura T Shiotsuki T Precocious metamorphosis in transgenic silkworms overexpressing juvenile hormone esterase Proc Natl Acad Sci USA 2005 102 11751 11756 10.1073/pnas.0500954102 16087883 PMC1187958

[B37] Hammock BD Abdel-Aal YAI Mullin CA Hanzlik TN Roe RM Substituted thiotrifluoropropanones as potent selective inhibitors of juvenile hormone esterase Pestic Biochem Physiol 1984 22 209 223 10.1016/0048-3575(84)90092-0

[B38] Prestwich GD Eng WS Roe RM Hammock BD Synthesis and bioassay of isoprenoid 3-alkylthio-1,1,1-trifluoro-2-propanones: potent, selective inhibitors of juvenile hormone esterase Arch Biochem Biophys 1984 228 639 645 10.1016/0003-9861(84)90033-X 6696451

[B39] Kuniyoshi H Baba K Ueda R Kondo S Awano W Juni N Yamamoto D lingerer, a Drosophila gene involved in initiation and termination of copulation, encodes a set of novel cytoplasmic proteins Genetics 2002 162 1775 1789 12524348 10.1093/genetics/162.4.1775 PMC1462391

[B40] Carmon A Topbas F Baron M MacIntyre RJ dumpy interacts with a large number of genes in the developing wing of Drosophila melanogaster Fly (Austin) 2010 4 117 127 20473031 10.4161/fly.4.2.11953

[B41] Kawamura K Shibata T Saget O Peel D Bryant PJ A new family of growth factors produced by the fat body and active on Drosophila imaginal disc cells Development 1999 126 211 219 9847235 10.1242/dev.126.2.211

[B42] Monteiro FA Perez R Panzera F Dujardin JP Galvao C Rocha D Noireau F Schofield C Beard CB Mitochondrial DNA variation of Triatoma infestans populations and its implication on the specific status of T. melanosoma Mem Inst Oswaldo Cruz 1999 94 Suppl 1 229 238 10677723 10.1590/s0074-02761999000700037

[B43] Dias Neto E Correa RG Verjovski-Almeida S Briones MR Nagai MA da Silva W Jr Zago MA Bordin S Costa FF Goldman GH Shotgun sequencing of the human transcriptome with ORF expressed sequence tags Proc Natl Acad Sci USA 2000 97 3491 3496 10.1073/pnas.97.7.3491 10737800 PMC16267

[B44] Glover DM Hames BD DNA Cloning 3, A Practical Approach, Complex genomes Oxford University Press; 1995

[B45] Ewing B Hillier L Wendl MC Green P Base-calling of automated sequencer traces using phred. I. Accuracy assessment Genome Res 1998 8 175 185 9521921 10.1101/gr.8.3.175

[B46] Chou HH Holmes MH DNA sequence quality trimming and vector removal Bioinformatics 2001 17 1093 1104 10.1093/bioinformatics/17.12.1093 11751217

[B47] Huang X Madan A CAP3: A DNA sequence assembly program Genome Res 1999 9 868 877 10.1101/gr.9.9.868 10508846 PMC310812

[B48] Tang Z Choi JH Hemmerich C Sarangi A Colbourne JK Dong Q ESTPiper--a web-based analysis pipeline for expressed sequence tags BMC Genomics 2009 10 174 10.1186/1471-2164-10-174 19383159 PMC2676306

[B49] Conesa A Gotz S Garcia-Gomez JM Terol J Talon M Robles M Blast2GO: a universal tool for annotation, visualization and analysis in functional genomics research Bioinformatics 2005 21 3674 3676 10.1093/bioinformatics/bti610 16081474

[B50] Labarga A Valentin F Anderson M Lopez R Web services at the European bioinformatics institute Nucleic Acids Res 2007 35 W6 11 10.1093/nar/gkm291 17576686 PMC1933145

[B51] Webb E Enzyme Nomenclature 1992. Recommendations of the Nomenclature Committee of the International Union of Biochemistry (NC-IUB) 1992 Academic Press

[B52] Ashburner M Ball CA Blake JA Botstein D Butler H Cherry JM Davis AP Dolinski K Dwight SS Eppig JT Gene ontology: tool for the unification of biology. The Gene Ontology Consortium Nat Genet 2000 25 25 29 10.1038/75556 10802651 PMC3037419

